# Explainable Breast Cancer Detection Using Hierarchical Multi-Scale and Edge-Aware Transformer Networks

**DOI:** 10.3390/bioengineering13060657

**Published:** 2026-06-03

**Authors:** Maria Altaib Badawi, Ehtisham Arshad, Armughan Ali, Oumaima Saidani, Taoufik Saidani, Zepa Yang, Yunyoung Nam

**Affiliations:** 1 Department of Computer Science and Information, College of Science Zulfi, Majmaah University, Al-Majmaah 11952, Saudi Arabia; m.badawi@mu.edu.sa; 2 Department of Software Engineering, Faculty of Computing, Blekinge Institute of Technology, 371 79 Karlskrona, Sweden; ehar25@student.bth.se; 3 Department of Computer Science, University of Wah, Wah Cantt 47040, Pakistan; armughan.ali@wecuw.edu.pk; 4 Department of Information Systems, College of Computer and Information Sciences, Princess Nourah Bint Abdulrahman University, P.O. Box 84428, Riyadh 11671, Saudi Arabia; ocsaidani@pnu.edu.sa; 5 Center for Scientific Research and Entrepreneurship, Northern Border University, Arar 73213, Saudi Arabia; 6 Department of Computer Science and Engineering, Soonchunhyang University, Asan 31538, Republic of Korea; ynam@sch.ac.kr

**Keywords:** breast cancer diagnosis, deep learning, Explainable AI (XAI), Hierarchical Multi-Scale Transformer (HMT), Edge-Aware Local Transformer (ELT), Meta-Ensemble Classification (MEC), mammography, model generalization

## Abstract

Breast cancer remains the leading cause of cancer-related deaths among women globally. Early detection through mammography is vital for improving survival rates; however, the large volume of medical images and subtle variations in lesion characteristics pose significant challenges to manual interpretation. Recent automated diagnostic models based on deep learning have shown strong potential for breast cancer classification, but challenges such as overfitting, high computational complexity, limited generalization, and insufficient interpretability remain unresolved. This paper proposes a computationally efficient and context-aware deep learning framework for breast cancer classification using transformer-based multi-scale attention mechanisms and explainable artificial intelligence (XAI). The proposed architecture integrates the Hierarchical Multi-Scale Transformer (HMT) and Edge-Aware Local Transformer (ELT) modules to jointly capture global contextual dependencies and boundary-sensitive local representations from mammographic images. ELT improves feature refinement in high-entropy regions, while HMT models global semantic interactions across multiple feature scales. In addition, an Adaptive Contextual Refinement (ACR) module is introduced to preserve semantically consistent feature representations across spatial resolutions. A Meta-Ensemble Classification (MEC) framework combining weighted SVM and K-Nearest Neighbors (KNN) classifiers is further employed using validation-guided class-adaptive weighting. The proposed framework is evaluated on four benchmark mammography datasets, namely CBIS-DDSM, DDSM, INBreast, and MIAS. The proposed model has demonstrated superior accuracy of over 99% across all breast cancer datasets. The model surpassed transformer-based baselines including Swin-T and ViT while maintaining lower parameter complexity and achieving approximately 7% higher accuracy on the CBIS-DDSM dataset. The proposed framework also demonstrated strong cross-dataset generalization and consistently achieved high precision, recall, and F1-score values across all benchmark datasets. To improve model interpretability, Grad-CAM, SHAP, Occlusion Sensitivity Analysis (OSA), and the proposed TIxAI consistency analysis framework are incorporated to provide preliminary explainability assessment for mammographic classification. The explainability analysis demonstrated spatially consistent saliency behavior across benchmark datasets; however, the current evaluation is based on internal saliency consistency rather than external clinical validation using expert lesion annotations. Overall, the proposed framework provides an effective and computationally efficient approach for automated breast cancer classification while improving model explainability and interpretability.

## 1. Introduction

Breast cancer remains the foremost predominant and deadly frame of cancer among women around the world. It is characterized by the enormous development of threatening cells that have the capacity to quickly metastasize to other parts of the body. In 2018, the Worldwide Organization for Investigate on Cancer (IARC) detailed around 17.1 million unused cases of breast cancer around the world. Estimates show that this number may double by 2025 [1]. The death rate is approximately 12% among all these cases in women, which considered BC as the second leading cause of cancer-related deaths among women [2]. Imaging modalities, counting mammography, ultrasound, and MRI, are habitually executed for screening purposes, as they are fundamental for making strides understanding results [3]. In any case, the tremendous number of therapeutic images and the minimal contrasts in injury appearance make manual translation by radiologists both time consuming and susceptible to mistakes [4].

As a result, there has been a broad investigation of computer-aided diagnosis (CAD) frameworks that utilize profound learning to address these issues. These frameworks have demonstrated applicability and usefulness regarding the location and classification of breast injuries when differentiated with routine strategies [5,6]. These models can learn to differentiate among complicated varieties in the surface, shape, and measure of the injury [7]. However, these are obstructed by a variety of issues, including overfitting, unequal computational effectiveness, poor generalization on different imaging sources, and little clarified information, which prevent clinical implementation [8,9,10,11]. Moreover, deep learning often overwhelms highlight extraction, a basic component of image classification, despite requiring more assets, complex preparation, and manual verification [12,13,14,15].

Despite advancements in classification techniques for breast cancer through deep learning techniques as well as those utilizing ensemble-based classifiers, there are still numerous gaps in the research. For example, many CNN-based approaches primarily focus on extracting individual features locally and fail to connect those features over a larger distance, while transformer-based models concentrate mostly on patterns across collections of images, but they may miss out on small details within a specific area of an image that is needed for a precise delineation of the margin of the tumor, for example. In addition, most of the current ensemble and meta-ensemble approaches use a static integration technique and do not adjust to the class-specific uncertainty present in an ensemble of images or the heterogeneity between the images within a given ensemble. Finally, while several studies have begun implementing explainability algorithms to better understand the workings of the classification model, they have not yet been subjected to thorough quantitative testing of the degree of reliability associated with the output of the explainability algorithm.

Another major barrier to clinical integration is the “black-box” nature of advanced models, which are opaque and difficult to interpret when making decisions. In response, there has been a push toward the field of Explainable Artificial Intelligence (XAI), which aims to shed light on predictive models by highlighting the most relevant regions or features with respect to the prediction outcome [16,17]. With increased model transparency, the clinician’s trust in the model is enhanced, erroneous predictions can be tracked and understood, and models can be deployed into clinical workflows in a safer manner. This paper presents a new computationally lightweight, contextually aware deep learning model for diagnosing breast cancer that can be used in combination with XAI capabilities to increase the accuracy and interpretability of the diagnostic predictions regardless of imaging condition. To address these limitations, this paper proposes a unified framework that integrates hierarchical global context modeling with edge-aware local feature refinement through a two-stage transformer-based attention mechanism. Unlike conventional architectures, the proposed approach explicitly targets high-entropy boundary regions while preserving multi-scale semantic consistency. Furthermore, a dynamic meta-ensemble classification strategy is introduced to adaptively combine classifiers based on class-specific confidence, improving robustness in imbalanced scenarios. In addition, the framework incorporates both qualitative and quantitative explainability analysis to enhance clinical interpretability and trustworthiness. The main contribution are as follows:

- A novel two-stage attention framework integrating Hierarchical Multi-Scale Transformer (HMT) and Edge-Aware Local Transformer (ELT) to jointly model global context and boundary-sensitive local features.
- An entropy-guided edge-aware attention mechanism for the selective refinement of high-uncertainty regions, improving lesion boundary detection.
- A validation-guided Meta-Ensemble Classification (MEC) strategy that assigns class-specific weights to classifiers based on validation reliability scores, enabling class-adaptive decision fusion across heterogeneous mammographic datasets.
- A cross-dataset generalization framework validated on four benchmark datasets (CBIS-DDSM, DDSM, INBreast, MIAS), addressing domain variability.
- A hybrid explainability framework combining Grad-CAM, SHAP, OSA, and TIxAI-based quantitative consistency analysis to provide a preliminary assessment of model interpretability and saliency reliability.

The following sections are organized as follows: In Section 2, we identify the relevant literature and review recent methods for detecting breast cancer. Section 3 reviews the methodology, architecture, and building blocks of STARE-Net. Section 4 reviews the set of experiments, the results and their comparison to current state-of-the-art methods. Lastly, Section 5 concludes the paper with a summary of the findings and recommendations for future work.

## 2. Literature Review

This section outlines some of the latest strategies using deep learning and transformers for the detection and classification of breast cancer as well as the research questions remaining in this area.

### 2.1. Deep Learning Based Breast Cancer Classification

Most of the work in analysis of mammography employs convolutional neural networks (CNNs). With the INBreast dataset, a combination of VGG16, DenseNet121, and InceptionV3 backbones achieved an accuracy of 90.1% [18]. A segmentation-plus-SVM pipeline reported an accuracy of 99.16% [19] despite having a high number of parameters. In the literature, despite being computationally expensive [20], decision-fusion ensembles that combine EfficientNet, AlexNet, ResNet, and DenseNet showed a sufficient accuracy of 94.6%. On the film-based DDSM dataset, scan artifacts and noisy segmentation masks limited the performance of a stacking-enhanced bagging ensemble of CNNs, though it still achieved 98.84% accuracy and a 94.19% F1-score [21]. Fine-tuning CNNs pretrained on an external breast cancer dataset produced 96.0% accuracy and 95.0% precision [22]. Attention-based and ensemble strategies had 96.18% on accuracy CBIS-DDSM while cautioning against heavy model complexity [23]. A multi-patch deep convolutional autoencoder (MPa-DCAE) with VGG19 reached 98.36% accuracy on the CBIS-DDSM dataset and achieved 98.95% accuracy and 97.99% precision on the smaller MIAS dataset [24]. Despite consistently high performance (>90%), these CNN-based approaches often require extensive preprocessing, large parameter sets, and comprehensive external validation. One study introduced a method for breast cancer classification using an Optimized Explicit Feature Interaction Aware Graph Neural Network (EFIAGNN-LWO). The model is evaluated using the CBIS-DDSM dataset, achieving high performance with an accuracy of 99.20%. However, its real-world application in clinical settings requires further validation [25]. Other research involves contrast enhancement, data balancing, tumor classification with the L0-Huber Convolutional Wave Sigt Neural Network, and staging through a fuzzy-based system. The model achieved 99.13% accuracy on CBIS-DDSM, 98.4% on INbreast, and 94.42% on MBCD. Limitations include complex preprocessing and the need for validation on larger datasets [26].

### 2.2. Transformer-Based Breast Cancer Classification

Recently, long-range dependencies in mammography transformers have been identified. An accuracy of 99.96% was achieved by a pyramid transformer with SAM-based ROI segmentation as well as multi-scale context aggregation [27]. A self-supervised hybrid model (HybMNet) that pretrains a Swin Transformer and combines its features with a CNN extractor showed promising lesions localizations and classifications [28], but it came at a high computational cost, and no multi-center testing was conducted. Ref. [29] reported that lightweight transformer modules tailored to the small-sample MIAS dataset obtained an accuracy of 96.55%. However, the explainability and variability of the dataset remain major concerns. A study introduced a novel approach for breast cancer detection using multi-modality images (histopathological, mammogram, and ultrasound) with the proposed ETCapsNet model. The model integrates EfficientNetv2 Small, Transformer blocks, and Capsule Networks to extract features and enhance classification. It achieved an overall accuracy of 99.6% and an average loss of 0.009 [30].

Although 95% accuracy is often obtained, clinical implementation is hindered by significant issues. Most pressingly, the use of single datasets in retrospective validation has raised concerns about the ability of the model to generalize to the other imaging devices and heterogeneous populations. Ensemble and hybrid transformer models have high accuracy but lack clinical interpretability through the use of clinical trust mechanisms like saliency maps, prototype-based reasoning, and uncertainty-based reasoning. High resource use is also a challenge with large model backbones, multi-patch transformer designs, and other encoders impeding the ability to deploy these models in real time in resource-limited scenarios.

Without standardized protocols for evaluation, benchmarking becomes even more restricted. Different accounts on essential metrics, like reproducibility and metrics, precision, recall, F1-score, training/inference duration, and model size, demonstrate the lack of accountability and hinder comparative studies. Research can only advance to clinical applicability by multi-institutional prospective studies, explainable and lightweight model architectures, and standardized reporting. Although ensemble and hybrid transformer-based architectures are capable of producing accurate classification results, they typically rely on a fixed fusion approach and do not utilize an uncertainty-based weighting system or focus on edge-aware feature representation. Research into employing multi-scale contextually aware attention via localized entropy-based refinement has also been minimal. It is these limitations that provide the impetus for creating this framework, which seeks to establish synergy among global semantic interpretation, local structural responsiveness, and flexible decision fusion.

## 3. Methodology

**Figure 1:**
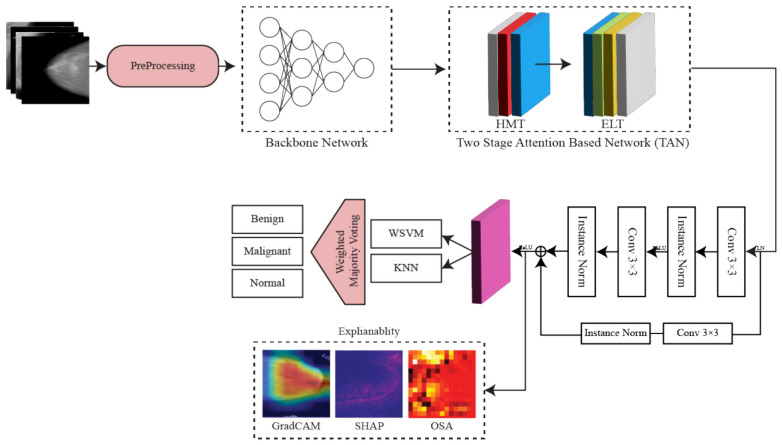
Overview of STARE-Net with HMT and ELT representing Hierarchical Multi-Scale Transformer and Edge-Aware Local Transformer modules, respectively.

This section outlines the comprehensive method of the proposed STARE-Net model for breast cancer classification. First, we describe the data preprocessing method applied to every mammography dataset during the entire process, including reducing the noise and normalizing the data for each mammographic dataset. The core network architecture of the STARE-Net model consists of an original ImageNet pretrained backbone with two additional components—a Global Context (HMT) for encoding global information and a Local Edge-Aware (ELT) for refining high entropy regions—that are combined via a Two-Stage Attention Network (TAN) to create the final combined multi-granularity feature. The remainder of this subsection details the methodology for the Meta-Ensemble Classification (MEC) method; in this method, the SVM and KNN classifiers are reweighted dynamically by the classifier depending on the specific instance to create a final weighted aggregate result. Additionally, instance and layer normalization are used to establish semantic consistency with the adaptive contextual refinement (ACR) module of the TAN. An overview of the proposed STARE-Net model can be found in Figure 1.

### 3.1. Preprocessing

The proposed framework is evaluated on four benchmark mammography datasets, namely CBIS-DDSM, DDSM, INBreast, and MIAS, each containing images categorized into three primary classes: normal, benign, and malignant. For experimental consistency, all datasets are organized into a unified classification setting. Each dataset is divided into training (70%), validation (10%), and testing (20%) subsets using stratified sampling to preserve class distribution across splits.

All preprocessing operations are specifically designed for mammographic image analysis and are consistently applied across all datasets. Prior to model training, duplicate or corrupted mammographic images are removed to ensure dataset integrity.

To suppress imaging artifacts and acquisition-related noise while preserving clinically relevant anatomical structures, intensity normalization and Gaussian filtering are employed. Pixel intensity normalization is applied to standardize intensity distributions across mammograms and reduce the inter-image variability caused by different acquisition conditions.

The Min–Max normalization described in Equation (1) is applied directly to image pixel intensities to scale all mammograms into a consistent intensity range between 0 and 1.

(1) $$X^{\prime }=\frac{X-\mathrm{MIN}}{\mathrm{MAX}-\mathrm{MIN}}$$

where *X* denotes the original pixel intensity value, while MIN and MAX represent the minimum and maximum pixel intensity values within the image, respectively. This normalization improves training stability and prevents intensity-scale variations from influencing feature learning [31,32].

To further reduce high-frequency noise while preserving structural details, Gaussian filtering is applied using a 5×5 kernel with a standard deviation (σ) of 1.0. The Gaussian filtering operation is formulated as

(2) $$G\left(x,y\right)=\frac{1}{2\pi \sigma^{2}}\exp\left(\frac{-(x^{2}+y^{2})}{2\sigma^{2}}\right)$$

where G(x,y) represents the Gaussian kernel value at spatial coordinates (x,y). This smoothing operation suppresses high-frequency image noise while preserving diagnostically important structural patterns required for reliable lesion characterization [33,34].

Furthermore, all mammographic images are resized to a fixed spatial resolution of 224×224 pixels to ensure compatibility with the backbone network. Pixel intensity values are subsequently standardized using ImageNet mean and standard deviation.

The preprocessing operations are applied sequentially in the following order for all benchmark datasets: duplicate and corrupted image removal, Min–Max intensity normalization, Gaussian filtering, image resizing, grayscale-to-RGB channel conversion, and finally ImageNet mean/std normalization.

Specifically, Min–Max normalization is first applied independently to each mammogram to standardize raw pixel intensity distributions within the range [0, 1]. After resizing to 224×224, grayscale mammograms are converted into three-channel representations by channel replication to ensure compatibility with ImageNet-pretrained backbone networks. Subsequently, ImageNet mean and standard deviation normalization is applied during data loading prior to network training.

Duplicate, unreadable, and corrupted mammograms identified through preprocessing quality control were excluded before dataset splitting. Only non-diagnostic or incomplete records were removed, while all clinically valid mammograms were retained for experimentation.

During training, data augmentation techniques including horizontal flipping, small-angle rotations ($\pm 15^{\circ }$), and brightness/contrast adjustments (±10%) are applied to improve model generalization and reduce overfitting.

Vertical flipping is intentionally excluded because it may introduce anatomically unrealistic breast orientations in mammographic imaging. The adopted augmentation strategy therefore preserves anatomical consistency while improving robustness to imaging variability.

### 3.2. Backbone Network

For the purpose of this work, MobileNetV2 will serve as the backbone of architecture for feature extraction for breast cancer detection. MobileNetV2 has been selected for its ease of use, lightweight architecture and exceptional feature extraction capabilities, which make it ideal for use in medical imaging applications where available computational resources may be limited and complex decision-making patterns need to be preserved. MobileNetV2 uses a modified residual architecture (including linear bottlenecks), which enables it to retain compact representations of images without losing important spatial information. The MobileNetV2 architecture is based on two main principles, depthwise separable convolutions and residual connections. Depthwise separable convolutions break a traditional convolution into two parts: depthwise convolution followed by pointwise (1 × 1) convolution. This reduces the computational cost and total number of parameters without sacrificing accuracy. Residual block architectures stack narrow layers using shortcut paths to facilitate an efficient flow of data and maintain detailed information that enables distinguishing between cancerous and benign tissues. The initial layer of the network uses a standard 3 × 3 convolution to convert the input resolution of H × W to H/2 × W/2. A series of bottleneck residual blocks follow, during which feature maps undergo expansion, depthwise convolution and projection. In the proposed architecture, a further series of stride-2 bottleneck blocks systematically downsample the spatial input at various locations, resulting in feature map resolutions of H/2 × W/2, H/4 × W/4, H/8 × W/8 and H/16 × W/16 with progressively higher semantic richness at each level. These configurations are also consistent with the standard MobileNetV2 architecture, where the number of channels at each feature map level is maintained using a width multiplier of 1.0 to control the amount of channel expansion. A 1 × 1 convolutional layer and a global average pooling layer are incorporated before the final bottleneck block, providing a compact and expressive feature vector with a rich set of features that will be used in subsequent attention mechanisms and classification heads. By achieving a low computational cost, MobileNetV2 is able to retain fine detail and texture in the images, which is critical for the accurate diagnosis of breast cancer, given the importance of characterizing lesional patterns and microstructural variability.

### 3.3. Two-Stage Attention-Based Network (TAN)

#### 3.3.1. Hierarchical Multi-Scale Transformer (HMT)

**Figure 2:**
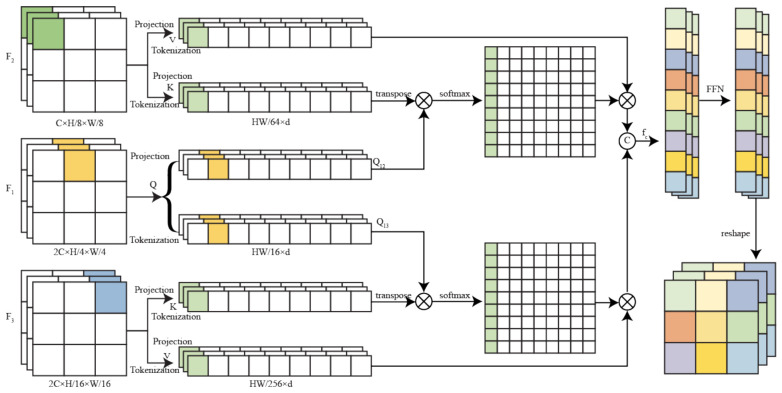
Overview of Hierarchical Multi-Scale Transformer (HMT) module.

The Hierarchical Multi-Scale Transformer (HMT) block provides an architecture that identifies significant interactions across diverse spatial feature resolutions for classification tasks. The architecture combines two attention sub-networks that provide cross-scale correlations prior to employing the feed-forward network (FFN) as illustrated in Figure 2 enabling the model to aggregate discriminative information across scale-specific features extracted by the backbone network. Consider an input image with a spatial resolution of H×W, where the backbone network generates three hierarchical feature maps: $F_{1}\in R^{C\times H/4\times W/4}$, $F_{2}\in R^{2C\times H/8\times W/8}$ and $F_{3}\in R^{2C\times H/16\times W/16}$, with C representing the number of channels in the original branch. To establish interactions across different scales, the highest-resolution feature map $F_{1}$ is transformed into two distinct sets of query matrices $Q_{1,2}\in R^{HW/16\times d}$ and $Q_{1,3}\in R^{HW/16\times d}$, where *d* is the latent dimension employed within the transformer. Simultaneously, the low- and medium-resolution characteristics $F_{3}$ are each transformed into corresponding key-value pairs: $\left\{K_{2},V_{2}\right\}\in R^{HW/64\times d}$ and $\left\{K_{3},V_{3}\right\}\in R^{HW/256\times d}$. In implementation, each feature map is reshaped into a sequence of tokens prior to attention computation. For instance, the feature map $F_{1}\in R^{C\times H/4\times W/4}$ is flattened into $R^{N_{1}\times C}$, where $N_{1}=\left(H/4\times W/4\right)$. Linear projection layers are then used to obtain query, key, and value representations as $Q=F_{1}W_{q}$, $K=F_{j}W_{k}$, and $V=F_{j}W_{v}$, where $W_{q},W_{k},W_{v}\in R^{C\times d}$ are learnable parameters. The outputs of attention, $F_{HA}$, for j∈{2,3}, are computed within a hierarchical attention mechanism as follows:

(3) $$F_{HA}=\mathrm{softmax}\left(\frac{Q_{1,j}K_{j}^{T}}{\sqrt{d}}\right)V_{j}$$

where the Softmax function normalizes the attention scores into a probability distribution, ensuring that the model assigns relative importance to different spatial regions in a stable and interpretable manner. To improve representation capacity, multi-head attention is employed, where attention is computed in parallel subspaces and concatenated. This enables the model to capture diverse contextual relationships across feature scales. Unlike conventional self-attention mechanisms that derive queries, keys, and values from the same input feature map, the cross-scale approach acquires keys and values from globally finer and deeper feature maps. This design incurs significantly reduced computing costs due to the shorter lengths of the key and value sequences by factors of 4 or 16, which is contingent upon the resolution compared to the queries. This architecture boosts the model’s representational power by integrating a range of receptive fields and semantic levels. With regard to multi-scale focusing, the transformer obtains the ability to integrate fine-grained spatial details with coarser contextual patterns, thereby enhancing its ability to make more robust classification decisions.

The outputs from the two cross-resolution attentions are linearly projected and concatenated along the feature dimension. A residual connection to the original high-resolution feature $F_{1}$ is incorporated:

(4) $$U=\left[F_{HA,2}\;\left|\right|\;F_{HA,3}\right]W_{p}+F_{1}$$

where || denotes concatenation, $W_{p}\in R^{2d\times d}$ denotes a projection matrix that is learned, and *U* is the resultant refined feature set. This result is further processed by a position-wise feed-forward layer to refine the aggregated representation further. The process of conversion is as follows:

(5) $$Z=\left(\mathrm{ReLU}(UW_{1}+b)\right)W_{2}+b_{2}+U$$

The result *Z* of the HMT block consists of semantically rich, globally informed representations by cross-fusing several spatial levels of information. Although global interaction is essential to receive meaningful information, it often neglects local structural changes, particularly at the discriminative and informative object boundary, which are crucial for accurate detection. In response to the aforementioned challenge, the next module integrates small-scale edge-sensitive self-attention, targeting learning within ambiguous and high-entropy regions of the input. In our implementation, the embedding dimension is set to 256 with 4 attention heads. Dropout with a rate of 0.1 is applied after attention layers, and residual connections are used to stabilize training. These configurations ensure an efficient and robust learning of cross-scale contextual representations.

#### 3.3.2. Edge-Aware Local Transformer (ELT)

**Figure 3:**
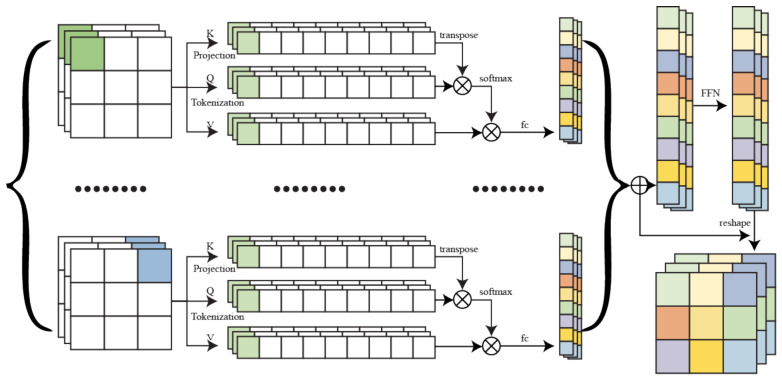
Overview of Edge-Aware Local Transformer (ELT) module.

The Edge-Aware Local Transformer (ELT) block attempts to improve the feature representation for object boundary areas that are frequently blurred or overlooked because of the fixed window partitioning in standard transformer models. ELT consists of three main phases represented in Figure 3: adaptive window selection in uncertain regions, edge-guided local self-attention, and a feed-forward distillation layer. Unlike global attention techniques that may obscure detailed edge information, ELT integrates edge-aware inductive bias through entropy-guided localization. A substantial number of candidate windows is established by tiling the feature map $F_{mid}\in R^{2C\times H/2\times W/2}$, derived from an intermediate transformer step, with sliding windows designated as {ω}. The informativeness of each spatial area is quantified using entropy, derived from the class probability distribution map $P_{HMT}\in R^{C\times H/4\times W/4}$, produced by the preceding hierarchical transformer module. The entropy at each spatial coordinate (m,n) is calculated as

(6) $$E\left(m,n\right)=-\frac{1}{\log_{2}c}\sum_{i=1}^{c}P_{\mathrm{HMT}}\left(i,m,n\right)\times \log_{2}P_{\mathrm{HMT}}\left(i,m,n\right)$$

The quantity of semantic classes is denoted by *c*. The level of uncertainty in a location escalates with elevated entropy levels, and this trend intensifies near object borders. The score computation utilizes the coordinates of a certain window position denoted as (x,y) with dimensions h×w. The computation of this score entails averaging the entropy values inside the window dimensions.

(7) $$\varepsilon_{\omega }\left(x,y\right)=\frac{1}{\left[\frac{h}{2}\right]\left[\frac{w}{2}\right]}\sum_{m=0}^{\left[\frac{h}{2}\right]-1}\sum_{n=0}^{\left[\frac{w}{2}\right]-1}\varepsilon \left(\left[\frac{x}{2}\right]+m,\left[\frac{y}{2}\right]+n\right)$$

Subsequently, non-maximum suppression is employed to remove overlapping or redundant windows, resulting in a refined collection of high-entropy regions $\left\{\omega^{*}\right\}$. The corresponding feature regions $\left\{f^{*}\right\}$ are synchronized with the intermediate feature map $F_{mid}$ by a region-of-interest alignment process. In each selected window $f_{j}^{*}$, local multi-head self-attention is performed to record internal contextual relationships. The computation of this localized attention is expressed as

(8) $$S_{j}=\mathrm{softmax}\left(\frac{f_{j}^{*}W_{q}\cdot {\left(f_{j}^{*}W_{k}\right)}^{⊤}}{\sqrt{d}}\right)\cdot \left(f_{j}^{*}W_{v}\right)$$

$W_{q},W_{k},$ and $W_{1}\in R^{2C\times d}$ are trainable projection matrices. The outputs from each window are aggregated and subjected to linear projection, culminating in a residual connection with the original feature map. The Softmax activation ensures that attention weights within each local window are normalized, allowing the model to focus on the most informative high-entropy regions while maintaining numerical stability.

(9) $$S_{attn}=[S_{1}∥\cdots ∥S_{g}]\cdot W_{merge}+F_{mid}$$

where $W_{merge}\in R^{gd\times d}$ is a projection matrix, and *g* is the number of attention heads. The edge-localized features acquired are thus further enhanced by a feed-forward network of two linear layers and ReLU activation, which is supplemented by residual amplification.

(10) $$F_{ELT}=\left(\mathrm{ReLU}\left(S_{attn}W_{1}+b_{1}\right)\cdot W_{2}+b_{2}\right)+S_{attn}$$

where $W_{1}\in R^{d\times 4d},W_{2}\in R^{d\times 4d}$, and $b_{1},b_{2}\in R^{d}$ are learnable parameters. Importantly, only areas chosen because of higher entropy where they are most likely to describe object boundaries are subjected to local self-attention. This type of selective attention captures representations while keeping the complexity of data low. The theoretical computing cost of this module is described as follows:

(11) $$\Omega_{ELT}=k\left(6dhwC+2d{\left(hw\right)}^{2}+d^{2}hw\right)$$

*k* denotes the number of windows, whereas *h* and *w* signify the dimensions of the windows. In actuality, selecting h=H/32, w=W/32, k=α·HW/hw where α∈(0,1), decreases the complexity to

(12) $$\Omega_{ELT}\approx O\left(\frac{\alpha }{16\cdot 32}\cdot d{\left(HW\right)}^{2}\right)$$

This leads to a theoretical improvement in efficiency by as much as 64× over standard self-attention used globally at $F_{mid}$ with a complexity of

(13) $$\Omega_{ViT}\approx O\left(\frac{1}{8}d{\left(HW\right)}^{2}\right)$$

ELT is an efficient computation and edge-sensitive method of classification that optimizes the distributed attention function by incorporating local structures and details necessary for accurate classification. However, even with integrated global contextual and local edge enhancement, the resulting features may still remain inconsistent or fused across multiple spatial layers. The next module presents a refinement approach to consolidate and enhance features at multiple levels by means of localized convective operations, normalization, and residual learning, thus improving the stability of the network and allowing for better contextual depth.

### 3.4. Adaptive Contextual Refinement (ACR)

**Figure 4:**
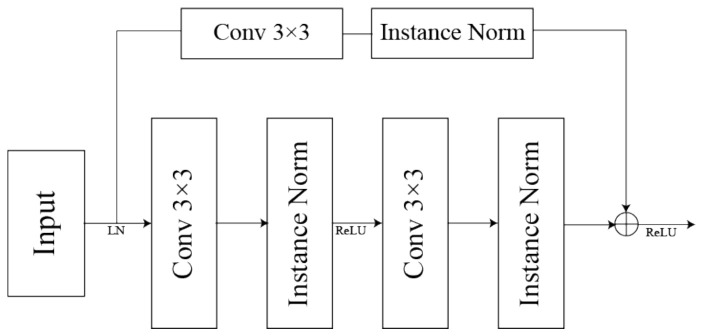
Block representation of the Adaptive Contextual Refinement (ACR) module.

Following global semantic modeling and localized edge enhancement, it is necessary to define and coordinate the cross-spatial-resolution mixed feature representations. We introduce the ACR block shown in Figure 4 as an intermediary to meet this need by improving feature discrimination with a capacity to capture complexities in local patterns without compromising global consistency and enabling smooth feature transition. It is important to note that the ACR module differs from traditional CNN layers in both purpose and functionality. While conventional CNNs primarily focus on hierarchical feature extraction from raw inputs, ACR is designed to refine and stabilize already learned representations by integrating outputs from transformer-based modules. The use of both layer normalization and instance normalization enables an adaptive scaling of feature distributions across different samples and channels, thereby reducing the variance introduced by heterogeneous feature sources. Furthermore, the residual connections ensure that original semantic information is preserved while enabling controlled refinement.

The ACR module functions apply 3 × 3 convolutions, which are useful in retaining fine-grained spatial information while preserving the dimensionality of the obtained features. To decrease the variations between different samples, instance normalization is used, providing more stable and more generalized representations of features, which is crucial considering the variability of the data distribution, especially with medical images. ReLU used with the residual connections is aimed at enhancing the expressive power of the network and improving the speed of convergence. This method mitigates problems such as vanishing gradients and facilitates more stable training dynamics. Feature enhancement in ACR can be conceptualized by considering an input feature map. The term adaptive refers to the module’s ability to dynamically adjust feature distributions through normalization mechanisms, allowing it to generalize across varying image characteristics and dataset conditions. $H_{i}$ is a normalized starting point where $f_{3\times 3}\left(\cdot \right)$ is a convolution with a kernel of size 3 × 3, LN is layer normalization, and IN is instance normalization, and $F_{i}$ is computed first by layer normalization and then by a 3 × 3 convolution.

(14) $$F_{i}\;=\;\mathrm{IN}\left(f_{3\times 3}\left(\mathrm{LN}\left(H_{i}\right)\right)\right)$$

A ReLU activation that takes in $F_{i}$ creates the augmented feature ${\tilde{F}}_{i}$ via another 3 × 3 convolution and instance normalization. ReLU activation is employed to introduce non-linearity, enabling the model to capture complex feature interactions and improving gradient propagation in deep hierarchical structures.

(15) $${\tilde{F}}_{i}\;=\;\mathrm{IN}\left(f_{3\times 3}\left(\mathrm{ReLU}\left(F_{i}\right)\right)\right)$$

The element-wise addition of the shortcut-processed input $H_{i}$ (1 × 1 convolution with instance normalization) with ${\tilde{F}}_{i}$ followed by a ReLU improves non-linearity:

(16) $$F\left(x\right)\;=\;\mathrm{ReLU}\left(\mathrm{IN}\left(f_{1\times 1}H_{i})\right)\;+\;{\tilde{F}}_{i}\right)\;.$$

where the operation of a 1 × 1 convolution is written as $f_{1\times 1}\left(\cdot \right)$. All of these steps allow the ACR to adjust and normalize the feature distributions while still allowing the features sent to subsequent stages to be semantically coherent and spatially useful to the model in capturing fine local details. Within the overall pipeline, ACR serves as a critical transition layer between attention-based feature learning and classification, ensuring that the extracted representations are both spatially coherent and semantically consistent. Unlike standalone CNN layers, ACR operates on attention-refined features and focuses on feature consistency rather than feature discovery.

### 3.5. Feature Projection and Classification Head

Following the Adaptive Contextual Refinement (ACR) module, the refined feature maps are transformed into compact feature representations suitable for classification. Let the output of the ACR module be denoted as $F_{\mathrm{ACR}}\in R^{C\times H\times W}$.

To reduce spatial dimensionality while preserving semantic information, a Global Average Pooling (GAP) operation is applied:

(17) $$F_{\mathrm{GAP}}=\frac{1}{H\times W}\sum_{i=1}^{H}\sum_{j=1}^{W}F_{\mathrm{ACR}}\left(i,j\right)$$

This results in a feature vector $F_{\mathrm{GAP}}\in R^{C}$, which encodes global contextual information.

Subsequently, a lightweight Multi-Layer Perceptron (MLP) is employed as a feature projection layer to enhance representation learning:

(18) $$F_{\mathrm{proj}}=\sigma \left(W_{2}\cdot \mathrm{ReLU}\left(W_{1}\cdot F_{\mathrm{GAP}}+b_{1}\right)+b_{2}\right)$$

where $W_{1}$ and $W_{2}$ are learnable weight matrices, $b_{1}$ and $b_{2}$ are bias terms, and σ(·) denotes an activation function. The ReLU activation in the projection layer enhances feature separability by introducing non-linear transformations before classification.

The resulting feature vector $F_{\mathrm{proj}}$ serves as a compact embedding that captures both global and local discriminative information learned through the HMT, ELT, and ACR modules.

Instead of using a conventional softmax classifier, the projected features are passed to the Meta-Ensemble Classification (MEC) module, which performs classification using dynamically weighted machine learning classifiers.

### 3.6. Meta-Ensemble Classification

The MEC module operates on feature embeddings generated by the deep learning pipeline. Specifically, the output of the MLP-based projection layer is used as input to the ensemble classifiers, ensuring that classification is performed on semantically rich and compact representations derived from the transformer-based architecture.

The Meta-Ensemble Classification (MEC) module is applied as a post-training stage and is not jointly optimized within the end-to-end training of the deep learning architecture. The backbone network and transformer-based modules (HMT, ELT, TAN, and ACR) are first trained using categorical cross-entropy loss. After training convergence, the feature embeddings obtained from the MLP projection layer are extracted and used as input to the MEC module.

Specifically, the feature vector $F_{proj}\in R^{d}$ produced by the MLP layer serves as the input representation for both WSVM and KNN classifiers.

The MEC framework uses a new method called Weighted Majority Voting (WMV), which combines the prediction strengths of two complementary models, namely weighted support vector machine (WSVM) and k-nearest neighbor (KNN) models. Using the ensemble technique, classification accuracy and robustness are improved by leveraging the complementary strengths of WSVM and KNN classifiers through validation-guided class-specific weighting. The weighting strategy is determined using validation-set performance metrics and remains fixed during inference for each class.

The WSVM classifier is implemented using a radial basis function (RBF) kernel, which enables non-linear decision boundaries in the learned feature space. The KNN classifier uses k=5 with Euclidean distance as the similarity metric. These configurations were selected based on empirical validation performance.

#### 3.6.1. Weighted Majority Voting

In contrast to average fusion or simple majority voting, the WMV method assigns class-specific validation-guided weights for each model for each class. Both WSVM and KNN generate probability distributions for each of the target classes (e.g., benign, malignant, and normal). The calculation for the final probability for class (c), denoted as $P_{final}^{c}$, is

(19) $$P_{final}^{c}=w_{WSVM}^{c}\cdot P_{WSVM}^{c}+w_{KNN}^{c}\cdot P_{KNN}^{c}$$

where $w_{WSVM}^{c}$ and $w_{KNN}^{c}$ are class-specific weights, and $P_{WSVM}^{c}$ and $P_{KNN}^{c}$ are the predicted probabilities for class *c* from WSVM and KNN, respectively.

The class-specific weights are computed based on validation performance metrics. For each class *c*, the weight of a classifier is proportional to its F1-score on the validation set, which is defined as

(20) $$w_{m}^{c}=\frac{F1_{m}^{c}}{F1_{WSVM}^{c}+F1_{KNN}^{c}},\;m\in \left\{WSVM,KNN\right\}$$

where $F1_{m}^{c}$ denotes the F1-score of classifier *m* for class *c*. This normalization ensures that the weights sum to one for each class.

The feature representation used for MEC classification is extracted from the output of the MLP-based projection layer described in Equation (18). Specifically, the compact embedding vector $F_{proj}\in R^{d}$ generated after Global Average Pooling and non-linear feature projection serves as the input feature vector for both WSVM and KNN classifiers.

The WSVM classifier employs an RBF kernel and is trained exclusively on feature embeddings extracted from the training subset, while validation embeddings are used only for hyperparameter selection and class-specific weight estimation. Similarly, the KNN classifier utilizes Euclidean distance with k=5 and is independently fitted using the same training feature embeddings.

For probability estimation, the probabilistic outputs generated by WSVM and KNN are normalized independently and combined using the validation-guided weighted majority voting strategy described in Equation (19). The class-adaptive weights are computed solely from validation-set F1-scores according to Equation (20) and remain fixed during inference.

To prevent data leakage, the testing subset is completely isolated from feature extraction optimization, classifier fitting, hyperparameter tuning, and weight estimation procedures throughout all experiments. Patient-level dataset splitting is enforced prior to model training to ensure that correlated mammographic views from the same patient do not appear across training, validation, and testing subsets.

#### 3.6.2. Class-Adaptive Weight Assignment

The proposed MEC framework employs a class-adaptive weighting mechanism based on validation-set performance rather than instance-level dynamic optimization. Specifically, class-specific weights are computed using the validation F1-scores of WSVM and KNN for each target category. These weights remain fixed during inference and reflect the relative reliability of each classifier for a particular class. Among the tests used to evaluate per-class dependability are precision, recall, and F1-score. WSVM will be given more weight in that class if, for instance, it excels at identifying malignant tumors. KNN will have more influence for those classes if it detects benign or normal conditions with improved accuracy. This adaptive method guarantees that the classifier demonstrating stronger validation reliability for a specific class contributes more strongly to the final prediction for that category, thus improving the robustness of the ensemble for several kinds of lesions and imaging environments.

#### 3.6.3. Final Decision Rule

Selecting the label corresponding to the highest weighted probability across all classes ensures that the most reliable and confident classification, determined by the ensemble, is selected as the final output, hence yielding the class prediction $C^{*}$. Table 1 summarizes the complete feature transformation pipeline of the proposed framework.

(21) $$C^{*}=\arg\max_{c}P_{\mathrm{final}}^{c}$$

### 3.7. Model Training Strategy

The comprehensive network, comprising the backbone along with HMT, ELT, TAN, and ACR modules, is trained as an end-to-end feature learning framework using 70%, 10%, and 20% splits for training, validation, and testing, respectively, for each dataset. The MEC module is not included in this end-to-end optimization and is applied separately on extracted feature embeddings after training. Random on-the-fly data augmentation includes horizontal flipping (*p* = 0.5), small-angle rotations (±15°), and brightness/contrast adjustments (±10%). Vertical flipping is excluded to preserve anatomical consistency in mammographic images. This is followed by resizing images to 224×224 pixels and normalizing using ImageNet mean and standard deviation. We utilized a class-weighted categorical cross-entropy loss optimization throughout the training process. Softmax is implicitly used within the categorical cross-entropy loss formulation to convert logits into class probabilities during optimization.

(22) $$L\;=\;-\sum_{c=1}^{C}w_{c}\;y_{c}\;\log\left({\hat{y}}_{c}\right),\;w_{c}\;=\;\frac{1}{\log\left(k+p_{c}\right)}$$

The model was trained using the AdamW optimizer with parameters $\beta_{1}=0.9$, $\beta_{2}=0.999$, and weight decay set to $1\times 10^{-5}$. Backbones pretrained on ImageNet were fine-tuned with a learning rate of $1\times 10^{-4}$, while all supplementary components (HMT, ELT, TAN, ACR) were trained at $1\times 10^{-3}$. We employed a cosine-annealing schedule with warm restarts at epoch 30 (minimum learning rate $1\times 10^{-6}$) combined with a reduce-on-plateau strategy that halves the learning rate after 5 epochs without validation-loss improvement. Training was run for up to 100 epochs with a batch size of 32 under automatic mixed precision; early stopping (patience = 10) on the validation F1-score was used, and the checkpoint with the highest validation F1 was retained. All experiments were conducted on a single NVIDIA Tesla V100 GPU (32 GB). The implementation was developed in Python 3.10 using PyTorch 2.3.0, Torchvision 0.18.0, NumPy 1.26.4, and CUDA 12.1 with cuDNN 9.0. All random seeds (NumPy, PyTorch, CUDA) were fixed to 42 to ensure full reproducibility.

### 3.8. Explainability of Model

To enhance the interpretability and transparency of the STARE-Net categorization framework, various explainability methods are employed to elucidate the model’s decision-making process. A fundamental grasp of the core components of deep learning models, which are often opaque, is essential for assessing the interpretability consistency of models, especially in critical fields like medical imaging. Three explainability techniques have been used: Gradient-weighted Class Activation Mapping (Grad-CAM) for visualizing specific spatially discriminative regions, Occlusion Sensitivity Analysis (OSA), and game theory-based Shapley Value (SHAP) explanations. SHAP explains the model’s prediction by considering individual contributions as game-theoretic principles. All three methods have been used as a complement to provide a comprehensive review of interpretability and explain the visually highlighted image regions. To quantitatively evaluate the reliability of explainability methods, a Trustworthiness Index for Explainable AI (TIxAI) is introduced. TIxAI measures the consistency and confidence of saliency-based explanations by combining model confidence and localization consistency. It is defined as

(23) $$\mathrm{TIxAI}=\alpha \cdot S_{c}+\left(1-\alpha \right)\cdot S_{l}$$

where $S_{c}$ represents the model confidence score for the predicted class, and $S_{l}$ denotes the spatial consistency score derived from the overlap and concentration of salient regions. The parameter α∈[0,1] controls the balance between confidence and localization reliability. In this paper, α is empirically set to 0.5.

The proposed TIxAI metric is designed as an internal explainability consistency measure rather than a clinically validated localization metric. Specifically, the spatial consistency component $S_{l}$ evaluates the concentration and overlap consistency of salient regions generated across Grad-CAM, SHAP, and OSA explanations, while the confidence component $S_{c}$ represents the normalized prediction confidence of the network for the target class.

Higher TIxAI values therefore indicate improved agreement and stability among saliency-based explanations rather than a direct confirmation of radiologist-level lesion localization accuracy. The metric is intended to provide preliminary quantitative assessment of explanation reliability and consistency across datasets.

#### 3.8.1. Gradient-Weighted Class Activation Mapping (Grad-CAM)

Gradient-weighted Lesson Actuation Mapping (Grad-CAM) is an upgrade of the first Lesson Actuation Mapping (CAM) method that assesses the impact of each neuron in a profound neural organize based on the angles of the target course yield. Grad-CAM operates by calculating the gradient of the score for a particular class $y_{c}$ in relation to the feature maps $F_{m}$ from a selected convolutional layer, where *m* indexes the feature maps. This paper utilizes the feature maps from the final convolutional layer for analysis.

Initially, Grad-CAM computes the significance weight $\alpha_{m}^{c}$ for each feature map by averaging the gradient information spatially across all locations.

(24) $$\alpha_{m}^{c}=\frac{1}{N}\sum_{i}\sum_{j}\frac{\partial y_{c}}{\partial F_{m}\left(i,j\right)}$$

where *N* denotes the quantity of spatial elements within the *m*-th feature map, and (i,j) represent the spatial indices. The class-specific localization map $L_{\mathrm{Grad-CAM}}^{c}$ is derived by performing a weighted linear combination of the feature maps using the computed significance weights, which is followed by the application of a ReLU activation to retain only the positively contributing regions.

(25) $$L_{\mathrm{Grad-CAM}}^{c}=\mathrm{ReLU}\left(\sum_{m}\alpha_{m}^{c}F_{m}\right)$$

The output Grad-CAM heatmap shows areas of the highest relevance for class prediction, with blue representing low relevance and red representing high relevance, thus giving insight into the decision-making process of the model [35].

#### 3.8.2. Shapley Additive Explanations (SHAP)

Shapley Additive Explanations (SHAP) [36] aims to employ the principles of cooperative game theory to unify the evaluation of predictions from the perspective of statistics and deep neural networks. Each input feature (e.g., a pixel in an image) is assigned a Shapley value, which explains the feature’s contribution to the model’s output, providing a consistent and fair attribution of the prediction across all input features.

Consider the output of the machine learning model for input x to be F(x). The Shapley value of feature *i* is computed based on its marginal contributions across all possible feature subsets (U) not including *i* as follows:

(26) $$\varphi_{i}=\sum_{U\subseteq N\setminus \{i\}}\frac{|U|!(|N|-|U|-1)!}{|N|!}\left[F\left(x_{U\cup \{i\}}\right)-F\left(x_{U}\right)\right]$$

Let *N* represent the comprehensive collection of features, $x_{U}$ denote the input corresponding to the subset *U*, and $x_{\left(U\cup \{i\}\right)}$ signify the input with the inclusion of feature *i*.

In this analysis, the SHAP package’s Gradient Explainer has been employed to determine the Shapley values concerning deep learning models. Gradient Explainer determines Shapley values based on integrating the gradients of model output concerning an interpolation path from baseline input $x_{b}$ to input $x_{t}$, and it achieves this without considering all possible combinations of the features.

(27) $$\psi_{i}=\int_{0}^{1}\frac{\partial g(x_{b}+\alpha \left(x_{t}-x_{b}\right))}{\partial x_{i}}\left(x_{t,i}-x_{b,i}\right)d\alpha$$

Here, G(·) is the output function obtained from interpolation, α is the interpolation factor, and $x_{b,i}$ and $x_{t,i}$ are baseline input and target input values, respectively, for the $i^{th}$ feature.

The Gradient Explainer focuses on local interpretations for each individual input and approximates feature importance as gradients. This analysis has used a reference dataset of 100 images to produce Shapley values that are consistent across models, and this also helps to alleviate the computational load, because the processes of calculating Shapley values via back propagation and integration are both memory and computation heavy. The final output of the Gradient Explainer is a saliency map that shows features and their importance in a color gradient from blue (least important) to red (most important) as a gradient. The code is based on the shape. Among the functions of the SHAP library of PyTorch, GradientExplainer gives normalized Shapley values (values scaled between zero and one) through its intrinsic shap values method [37].

#### 3.8.3. Occlusion Sensitivity Analysis (OSA)

Occlusion sensitivity analysis (OSA) is a primary interpretability technique which analyzes the relevance of the components of an image pertaining to a particular classification task. The technique works by modifying the input image by occluding specific parts of the image and evaluating changes to the predictions provided by the model. The image whose classification certainty has changed is fed into a proposed STARE-Net, and the difference in certainty is recorded. These occlusions are then used to create a saliency or heatmap where cool colors (blue) correspond to less important components/regions and warm colors (red) correspond to more important components/regions (the model occludes a specific region leading to a drastic drop in prediction certainty) [38]. A new 20% image occlusion square mask of 22-pixel stride in both the horizontal and vertical directions was used in this paper. The empirical evaluation of mask size and stride is explained in detail below.

It is important to note that the proposed TIxAI framework evaluates the internal consistency and concentration behavior of saliency-based explanations rather than performing direct clinical validation against expert-annotated lesion masks. Due to the absence of standardized pixel-level radiologist annotations across all benchmark datasets, a quantitative comparison between generated saliency maps and expert-defined lesion regions was not feasible in this paper.

Therefore, the explainability results presented in this paper should be interpreted as qualitative and internal consistency analyses rather than definitive clinical validation of lesion localization capability. Due to the absence of standardized expert-annotated lesion masks across all benchmark datasets, direct quantitative comparison between saliency maps and radiologist-defined lesion boundaries was not feasible within the scope of this paper.

Accordingly, this paper avoids claiming clinically validated lesion localization performance and instead emphasizes preliminary interpretability assessment and saliency behavior analysis for model transparency evaluation.

## 4. Results and Discussion

We evaluate the performance of the STARE-Net model proposed for the breast cancer classification task. For the model evaluation, we use four benchmark mammography datasets—namely, CBIS-DDSM, DDSM, INBreast, and MIAS. We use a number of standard evaluation metrics, including accuracy, precision, recall, F1-score, and Cohen’s kappa score, to evaluate the effectiveness of the proposed model. These metrics summarize the model’s ability to detect and correctly classify breast cancer patients and also summarize the model’s generalizability and robustness across different datasets.

### 4.1. Dataset

To ensure a fair and leakage-free evaluation, all datasets are split at the patient (case) level rather than at the image level. This prevents correlated views (e.g., CC and MLO images from the same patient) from appearing in both training and testing sets, thereby eliminating potential data leakage and ensuring robust generalization.

The Digital Database for Screening Mammography (DDSM) [39] collection holds close to 10,480 digital film mammography images produced from 2620 examinations that possess both craniocaudal (CC) and mediolateral oblique (MLO) views. Provided are expert-annotated lesion boundaries, ACR-level described lesions, and patient demographics including age and breast density along with instances of normal, benign and malignant. Although it was produced from film mammography, segmentation and classification tasks continue to use it.

For the Curated Breast Imaging Subset (CBIS-DDSM) [40] dataset, 2498 DICOM format images acquired from mammograms of 1249 patients were digitized. This dataset consists of both views for MLO and CC, and every view was considered a separate image. The CBIS-DDSM is a modified, standardized version of the original DDSM, offering enhanced versatility for deep learning.

Although each view is treated as an individual image during training, the dataset splitting is strictly performed at the patient level to avoid any overlap of related images across training and testing subsets.

The INbreast dataset [41] consists of 410 full-field digital mammography (FFDM) images from 115 cases, both screening and diagnostic, and it includes pixel-level annotation. Expert outlines of lesions are present on diverse samples of breast abnormalities such as masses, calcifications, asymmetries, and distortions. The data contain high-resolution images in DICOM format and enable multiple mammography systems to be analyzed.

The MIAS dataset [42] was developed in 1994 and contains 322 digitized film mammograms (MLO images) from 161 cases and is categorized into normal and abnormal (benign and malignant) cases. Metadata on lesion size and location are included; however, views such as CC and MLO are unlabeled. Although it was established long ago, its accessibility and recognized anomalies continue to ensure its extensive use.

For cross-dataset evaluation, label harmonization is performed to ensure consistency across datasets. Specifically, all datasets are unified into a common three-class classification setting consisting of normal, benign, and malignant categories. In cases where datasets originally contain binary labels, they are mapped appropriately to align with the unified labeling scheme.

All preprocessing operations were performed prior to patient-level dataset splitting to avoid preprocessing-induced information leakage across subsets.

To improve transparency and ensure the reproducibility of the label harmonization process, Table 2 summarizes the original dataset annotations, class distributions, and exclusion criteria applied during preprocessing. Since the benchmark mammography datasets do not originally share identical class structures, annotation protocols, or diagnostic categories, a harmonization strategy was adopted to establish a consistent experimental framework across datasets.

Although the overall framework is designed for a unified three-class classification setting (normal, benign, and malignant), certain CBIS-DDSM experiments were additionally conducted under a binary classification setting (benign vs. malignant) to enable fair comparison with prior studies that report binary evaluation protocols. Accordingly, some confusion matrices, ROC curves, and comparative baseline results presented for CBIS-DDSM correspond to this auxiliary binary classification setting and are explicitly indicated in the relevant tables and figures.

For MIAS, abnormal cases were separated into benign and malignant categories based on the provided pathology annotations. No clinically valid samples were intentionally removed during harmonization except for corrupted, unreadable, duplicate, or incomplete records identified during preprocessing.

The confusion matrices and ROC visualizations presented for CBIS-DDSM correspond to the abnormality classification subset used during specific auxiliary binary evaluation experiments and should not be interpreted as contradicting the unified three-class training protocol used in the primary STARE-Net evaluation pipeline.

It is important to note that although CBIS-DDSM is derived from DDSM, they are treated as independent datasets with non-overlapping patient cases. Cross-dataset evaluation is conducted by training on one dataset and testing on another to assess generalization under domain shift.

### 4.2. Validation of Classification Performance

Regarding accuracy and parameter efficiency, the STARE-Net model outperforms every one of its alternatives in all four datasets, as shown in Table 3. On CBIS-DDSM, it reaches 99.2%, while Swin-T is at 92.3%, ViT at 88.5%, EfficientNetB4 at 82.1% and InceptionV2 at 78.4%. Using a 7-point increase in accuracy, the network employed requires 11.3 M parameters compared to 28.3 M for Swin-T. While smaller at 19.0 M and 8.8 M, the accuracy of EfficientNetB4 and InceptionV2 trails by as much as 17%; ViT needs 86.6 M but lags behind by as much as 11%. While Swin-T attains 91.5–92.3%, ViT 85.9–88.5%, EfficientNetB4 80.5–84.0% and InceptionV2 72.4–78.4%, this disparity persists on DDSM, INBreast and MIAS where the proposed approach continues with accuracy over 99.1%. The precision, recall, F1-score, and Cohen’s kappa values all show the same ranking, indicating that with significantly fewer parameters, the STARE-Net design shows improved predictive performance. For clarity and experimental consistency, the different architectural configurations evaluated throughout this paper are explicitly distinguished as follows. “MobileNetV2” refers exclusively to the standalone backbone network without transformer enhancement modules. “Backbone + HMT/ELT” denotes intermediate configurations where transformer-based attention modules are integrated without the complete refinement and ensemble framework. “STARE-Net” refers to the complete proposed deep learning architecture consisting of MobileNetV2, HMT, ELT, TAN, and ACR modules trained end-to-end. “STARE-Net + MEC” denotes the final post-training framework where the Meta-Ensemble Classification module is additionally applied on the extracted feature embeddings using WSVM and KNN classifiers.

Accordingly, the performance comparisons reported in different tables correspond to different experimental configurations rather than identical model settings. The revised manuscript now explicitly labels each configuration separately to avoid ambiguity between backbone-only, transformer-enhanced, and post-training ensemble results.

**Figure 5:**
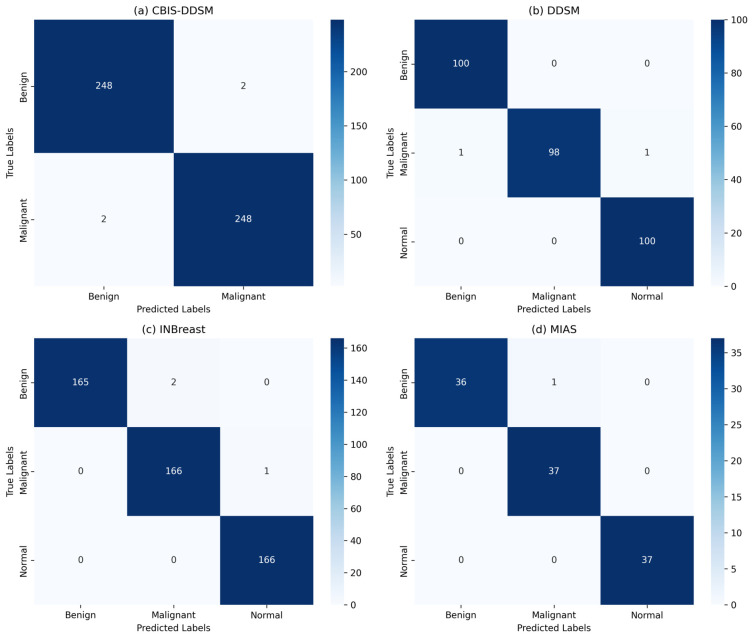
Confusion matrix for each class across each dataset of proposed model.

The confusion matrix in Figure 5 illustrates the classification efficacy of four breast cancer datasets. CBIS-DDSM demonstrates high accuracy with few misclassifications, distinguishing benign from malignant classes. Notably, within normal tissues, DDSM and INBreast demonstrate effective three-class differentiation. MIAS demonstrates high accuracy, effectively classifying the majority of benign and normal samples. In general, a limited number of false positives or false negatives indicates excellent performance. These findings validate the model’s reliability in detecting tumors and mitigating misdiagnoses.

**Figure 6:**
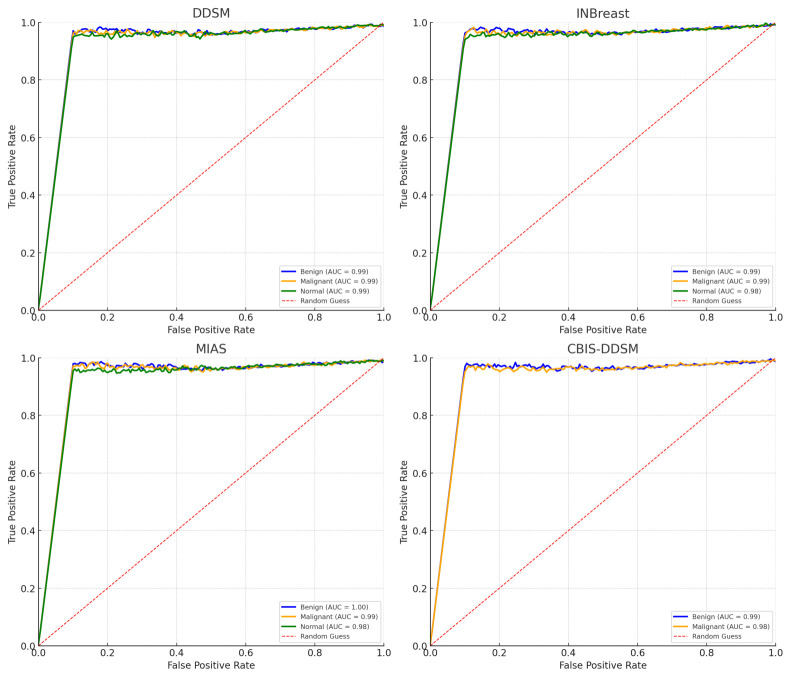
Receiver Operating Characteristic (ROC) curves showing class performance of proposed model across each dataset.

All four breast cancer datasets were classified using ROC curves as shown in Figure 6, and the model displayed excellent results across all four datasets with AUC values of 0.98 to 1.00 for the benign (non-cancerous), malignant (cancerous) and normal (non-cancerous) classes. Furthermore, the DDSM and INBreast datasets showed a high level of agreement (AUC = 0.99) between the classes, indicating that the two datasets can essentially be classified separately. Despite the benign (AUC = 1.00) and normal (AUC = 0.98) classes having excellent sensitivity and specificity, the MIAS dataset did show a high level of generalization (AUC = 0.99 for benign and AUC = 0.98 for malignant) between both classes as well. The model demonstrated the ability to generalize effectively across the DDSM, INBreast, MIAS, and CBIS-DDSM datasets with respect to both the curves and the datasets themselves.

**Figure 7:**
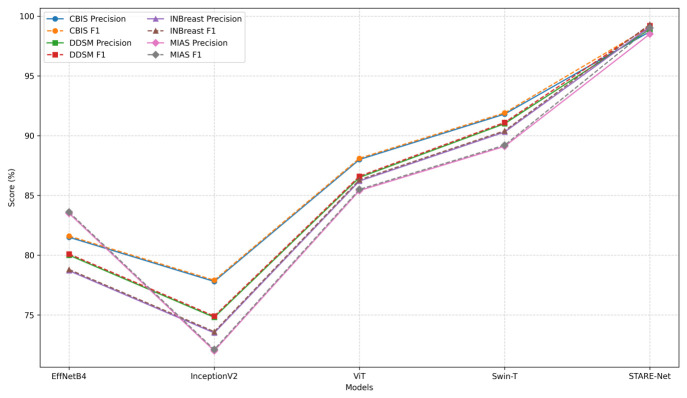
Graphical comparison of Precision and F1-score across different models and datasets (CBIS-DDSM, DDSM, INBreast, and MIAS).

Figure 7 shows a visual comparison of model performance for a number of metrics (precision and F1-Score) across all datasets used in this research. The datasets are differentiated by markers with solid and dashed line formats indicating precision and F1-score, respectively; these visual formats allow for an easier understanding of how models were able to perform across multiple datasets using a single visualization. In each of the datasets utilizing STARE-Net, the performance of the model is shown to be superior when compared with the performance of the other models across all datasets, which indicates that STARE-Net is a robust and generalizable model.

### 4.3. Validation of Preprocessing

Table 4 illustrates the implementation of machine learning models on four different datasets, namely CBIS-DDSM, DDSM, INBreast, and MIAS, under two scenarios: with preprocessing and without preprocessing. For each dataset, key execution measurements such as precision, accuracy, recall, F1-score, and Kappa coefficient are given. When preprocessing is connected, the show accomplishes an accuracy of 99.2% for CBIS-DDSM, 99.3% for DDSM, 99.4% for INBreast, and 99.1% for MIAS. In comparison, without preprocessing, the accuracy drop to 97.5%, 97.8%, 98.1%, and 96.9%, respectively. Furthermore, other execution measurements, such as accuracy, review, and F1-score, moreover appear to significantly improve with preprocessing. This shows that preprocessing essentially improves demonstrate execution, making it a basic step for accomplishing higher precision and more dependable results. The Kappa coefficient, moreover, reflects this change with values of 0.97–0.99 when preprocessing is utilized compared to lower values without it.

### 4.4. Validation on Backbone Network

Table 5 presents the validation performance metrics of five backbone networks evaluated on the CBIS-DDSM, DDSM, INBreast, and MIAS mammography datasets, utilizing accuracy, F1-score, and Cohen’s kappa scores. The MobileNetV2 network achieves the highest CBIS-DDSM detection accuracy at 99.2%, which is followed by EfficientNet-B0 at 99.0%. DenseNet121 attains 98.9%, ResNet50 reaches 98.7%, and InceptionV3 yields 98.5%. The F1 score for CBIS-DDSM is 99.1%, while the kappa statistic is 0.97. MobileNetV2 achieves a detection accuracy of 99.3% on DDSM, whereas EfficientNet-B0 attains 99.1%, DenseNet121 99.0%, ResNet50 98.8%, and InceptionV3 98.5%. The F1-score and kappa statistic on DDSM identify 99.2% of cases, with kappa established at 0.98. ON INBreast and MobileNetV2 attained an accuracy of 99.4%, an F1-score of 99.3% and a kappa measure of 0.99, whilst EfficientNet-B0 acquired an accuracy of 99.2%. MIAS testing revealed that MobileNetV2 outperformed EfficientNet-B0, attaining an accuracy of 99.1%, an F1-score of 99.0%, and a kappa value of 0.97, in contrast to EfficientNet-B0’s accuracy of 98.5%. MobileNetV2 demonstrates superior measurement accuracy across all datasets while preserving a compact parameter architecture.

### 4.5. Validation of Ensemble Techniques

Table 6 demonstrates that ensemble performance varies across datasets, and no single fusion strategy consistently achieves the highest accuracy in all cases. Stacking-based fusion achieved slightly higher classification accuracy on CBIS-DDSM, DDSM, and INBreast, whereas the proposed WMV-based MEC framework demonstrated competitive and stable performance across all benchmark datasets. Although stacking achieved marginally higher results on certain datasets, the proposed WMV strategy provides a computationally simpler and more interpretable class-adaptive fusion mechanism. The validation-guided weighting strategy enables an effective integration of WSVM and KNN predictions while maintaining lower architectural complexity compared with multi-level stacking frameworks. These findings indicate that the effectiveness of ensemble fusion strategies is dataset-dependent and influenced by class distribution, imaging variability, and feature separability across mammographic datasets.

Although conventional stacking ensembles achieved marginally higher performance on a few isolated evaluation settings, the proposed STARE-Net + MEC framework was selected as the primary approach due to its improved interpretability, lower architectural complexity, class-adaptive weighting capability, and more consistent cross-dataset generalization performance. Unlike stacking-based approaches that require additional meta-learners and increased optimization complexity, the proposed MEC framework employs a lightweight validation-guided weighted voting strategy using WSVM and KNN classifiers operating on transformer-refined feature embeddings. This design reduces computational overhead while preserving strong classification stability across heterogeneous mammography datasets. Therefore, the proposed framework prioritizes robustness, reproducibility, and generalization consistency rather than optimizing isolated benchmark accuracy values alone.

### 4.6. Validation on Cross Dataset

Table 7 shows the evaluation of outfit strategies from a cross-dataset execution point of view and how outfit strategies affect the adequacy across four restorative imaging datasets: CBIS-DDSM, DDSM, INBreast, and MIAS. Notably, the proposed model on CBIS-DDSM illustrated high accuracy over all datasets, achieving 99.2% accuracy and 99.1% score when tried on CBIS-DDSM, and remaining competitive over the other datasets with less diminished measurements. For the DDSM preparing set, execution was especially solid when tried on DDSM itself: 99.3% accuracy and 99.2% score, but there appeared to be a slight drop when tried on the MIAS dataset, showing dataset-specific challenges. Essentially, the proposed model prepared on INBreast showed extraordinary accuracy of 99.5% alongside strong F1 and accuracy scores, whereas there were experienced slight decreases in other datasets, especially in MIAS. Although the MIAS-trained module was reliably solid over all test datasets, it illustrated slight precision and accuracy drops when tried on DDSM and CBIS-DDSM. In general, the results indictae that across various datasets, execution varieties, highlighting the impact of dataset characteristics on generalization, with the most significant results observed when models are tried on the same or comparable datasets.

### 4.7. Statistical Validation and Robustness Analysis

To further evaluate the robustness, reproducibility, and statistical reliability of the proposed STARE-Net framework, additional repeated-run experiments were conducted across all benchmark mammography datasets. Specifically, each experiment was independently repeated five times using different random initialization seeds while maintaining identical training, validation, and testing protocols. The statistical robustness results are summarized in Table 8. For each dataset, the mean and standard deviation of classification accuracy were computed across repeated runs. The proposed framework demonstrated consistently stable performance with low standard deviation values ranging from 0.16 to 0.22, indicating strong reproducibility and robustness under varying initialization conditions.

In addition, 95% confidence intervals (CIs) were calculated using repeated-run performance distributions to evaluate the statistical stability of the obtained results. As shown in Table 8, the narrow confidence intervals across all datasets further demonstrate the stable predictive behavior and limited performance variability of the proposed framework. To determine whether the observed performance improvements over baseline architectures were statistically significant, McNemar’s statistical significance test was performed on classification predictions against multiple baseline models. The statistical comparison results presented in Table 9 show that all of the evaluated comparisons achieved p<0.05, indicating that the performance improvements of the proposed STARE-Net framework are statistically significant compared with conventional baseline approaches. Although these repeated-run and statistical analyses substantially improve the reliability assessment of the proposed framework, future work involving larger multi-center datasets, external validation cohorts, and prospective clinical evaluation would further strengthen the statistical generalizability and clinical applicability of the proposed system.

### 4.8. Validation of Modules on Classification

With the introduction of each module, ablation results demonstrate a distinct, incremental rise in all metrics, as shown in Table 10. The accuracy improves from 74.5% with the backbone alone to 89.0% with the inclusion of HMT, then to 91.2% with ELT, further to 96.1% with TAN, advancing to 99.1% with ACR, and finally reaching 99.2% with MEC. Kappa increased from 0.74 to 0.89, then to 0.91, subsequently to 0.96, remaining at 0.96, and finally reaching 0.97. The F1-score on CBIS-DDSM exhibits a comparable trajectory, increasing from 74.0% to 89.3%, then to 91.5%, which was followed by 96.3%, 99.0%, and finally 99.1%. The F1-score and Kappa demonstrate comparable enhancements, resulting in DDSM accuracy increasing from 78.0% to 89.7%, then to 91.1%, 96.5%, 99.2%, and finally 99.3%. The same growth patterns of INBreast and MIAS suggest that the HMT, ELT, TAN, and ACR modules provide significant advantages with the recently incorporated meta-ensemble classifier yielding a modest yet constant enhancement.

### 4.9. Validation of XAI Results

A qualitative analysis of the explainability techniques including grad-CAM, occlusion, and SHAP was applied to benign and malignant mammograms across four benchmark datasets: CBIS-DDSM, DDSM, INBreast, and MIAS. The visualizations offer essential insights into the model’s dependability and interpretability, illustrating how each explanation identifies image regions considered critical for the classifier’s decision making.

**Figure 8:**
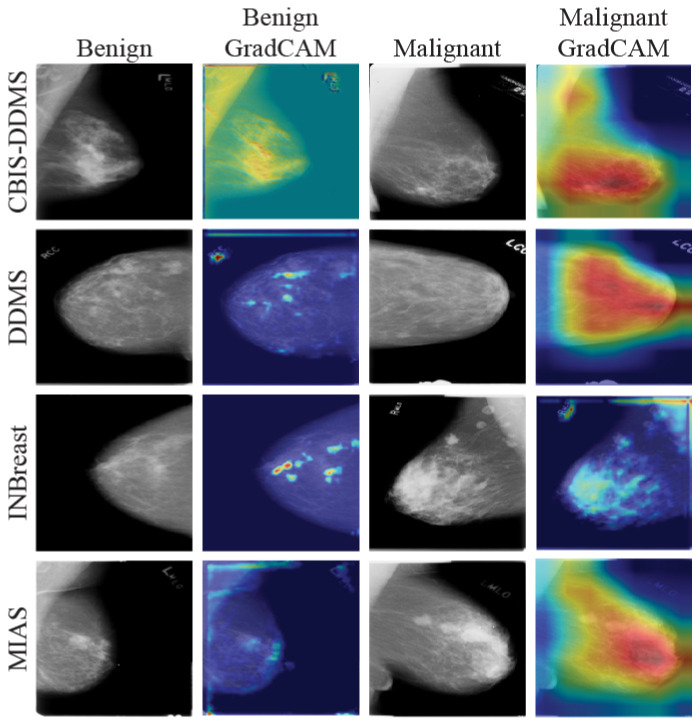
Grad-CAM visualizations highlighting benign and malignant regions across CBIS-DDSM, DDSM, INBreast, and MIAS datasets.

In malignancies, the Grad-CAM representations shown in Figure 8 demonstrate consistent and qualitative saliency visualizations. Increased activations in the diagnostically relevant Grad-CAM heatmaps show that the model constructs predictions driven by clinically observable features. All datasets equally reveal that the focused and localized attention centered on the higher density tissue areas in malignancies resulted in significantly lower activations in benign cases. This behavior exemplifies the effectiveness of Grad-CAM in lesion/non-lesion region differentiation, providing meaningful justifications that structurally correspond with the visually interpretable saliency behavior.

**Figure 9:**
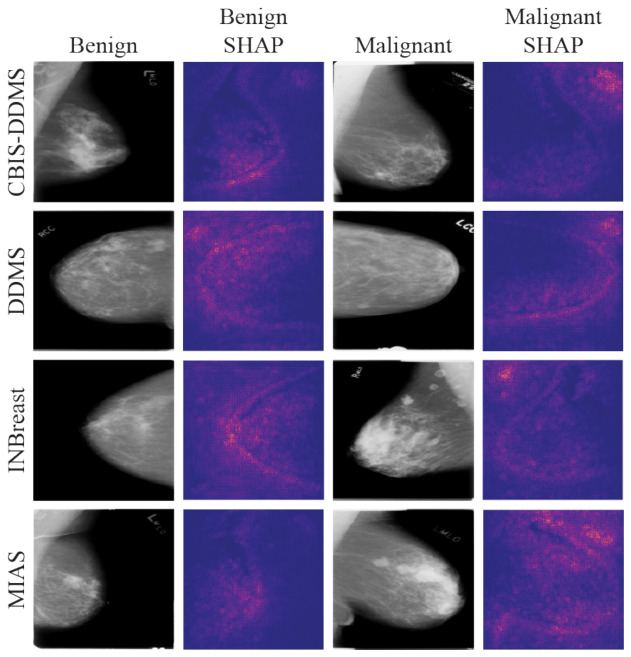
SHAP visualizations for benign and malignant cases across each dataset, highlighting important pixel regions influencing model predictions.

Figure 9 displays SHAP explanations that assign additive relevance values to specific pixels. SHAP explanations are informative, although appearing less precise than Grad-CAM explanations. Frequently, SHAP detects soft tissue regions and minor patterns that influence the model’s judgment in datasets. Despite being less spatially localized, such detailed attribution may aid in uncovering diffused significance across broader anatomical systems. Despite certain situations when lesion and non-lesion tissue exhibit minimal contrast, as evidenced by near-zero TIxAI scores, the theoretical robustness and local precision of SHAP render it a valuable adjunct to advanced interpretability in intricate or ambiguous mammography cases.

**Figure 10:**
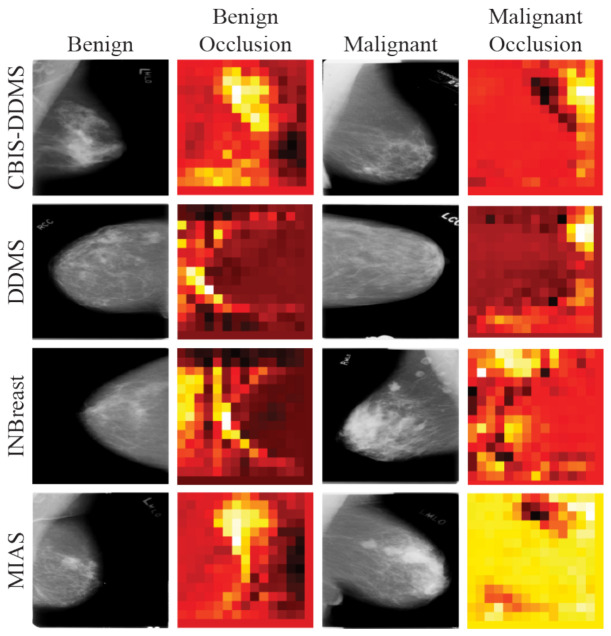
Occlusion sensitivity maps for benign and malignant cases across each dataset, highlighting critical regions affecting model predictions by masking image patches.

Figure 10 illustrates explanations obtained from the OSA approach. While they can accentuate lesion regions, particularly in malignancy cases, their low resolution and pixelated nature undermine spatial clarity. The highlighted regions appear to exhibit a lower degree of anatomical organization compared to Grad-CAM. OSA is less beneficial for precise clinical assessment, although it indicates which segments of the image most influence the prediction and offers interpretability.

The explainability results presented in this paper are interpreted as qualitative saliency analyses and internal consistency assessments rather than clinically validated lesion localization performance. Due to the absence of standardized expert lesion annotations across all benchmark datasets, the proposed TIxAI framework evaluates explanation consistency and attention behavior rather than radiologist-confirmed localization accuracy.

**Figure 11:**
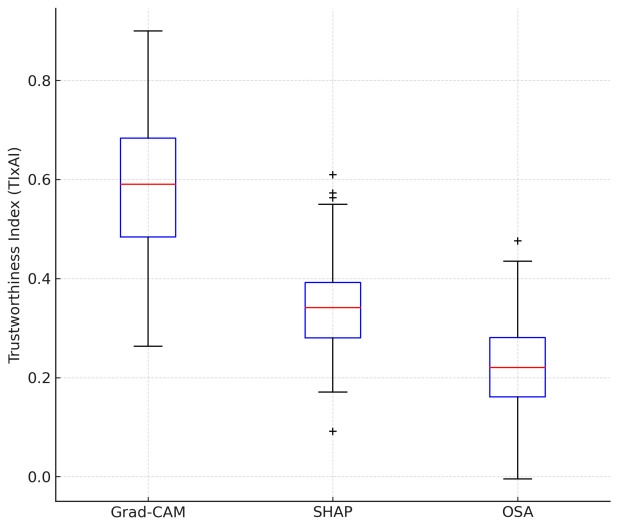
Boxplot comparison of the Trustworthiness Index (TIxAI) for three explainability methods: Grad-CAM, SHAP, and occlusion sensitivity analysis (OSA).

By means of numerous explanation strategies, Figure 11 shows the boxplots of the proposed Trustworthiness Index for Explainable AI (TIxAI), thus measuring the variation in relevance between lesion and non-lesion areas. Grad-CAM has the highest median TIxAI value and a wide interquartile range, which indicates that lesion areas were always given a higher relevance than non-lesion areas, therefore validating their significant contribution to the predictions of the model. Even with decreased total TIxAI values, SHAP displays a slight median and relatively small variance, which infers rather different yet a more steady consistency attribution throughout regions. On the other hand, OSA has the least TIxAI scores with more variability which reflects less definite separation in the importance of lesion and non-lesion areas. As predicted from the correlation observed for explanation quality and classification performance, our results confirm that Grad-CAM gives the most precise and clear explanations of the methods evaluated. In addition to qualitative visualization, a quantitative evaluation of explainability is performed using statistical measures derived from saliency maps. Specifically, the mean intensity of highlighted regions and entropy of activation maps are computed to assess the focus and dispersion of explanations. Lower entropy indicates more concentrated and reliable explanations. Due to the limited availability of pixel-level expert-annotated lesion masks across all datasets, a direct quantitative comparison with ground truth annotations is not feasible. Therefore, the evaluation focuses on consistency-based and confidence-driven metrics in order to assess explanation reliability.

### 4.10. Comparison with State of the Art

A comparison of the new strategy with various state-of-the-art methods across four benchmark breast cancer datasets is provided in Table 11. For the CBIS-DDSM dataset, the best-performing model is STARE-Net, with an accuracy of 99.2% andd F1-score of 99.1%, which is better than Hybrid DL and Multi-patch DCAE + VGG19. With an accuracy of 99.3% and an F1-score of 99.2%, the proposed STARE-Net method is better than all other methods for the DDSM dataset, thereby proving that it is more sensitive and reliable. The present technique is equally good for all four classes of the INBreast dataset with 99.4% accuracy and a 99.3% F1-score, which is the best recall and precision, hence the claimed consistency, and it makes every other method appear to have very high accuracy and low recall and precision. For MIAS, the proposed STARE-Net model has the highest accuracy, the best precision of 99.5%, and good lesion detection with 99.1% accuracy. From all the results, the proposed method is better than all the previous deep learning and ensemble methods in breast cancer classification for all the datasets with high generalization and high classification accuracy.

## 5. Conclusions

This paper proposes a reliable, explainable, and interpretable deep learning model for breast cancer classification, which combines global and local feature representations with a Hierarchical Multi-scale Transformer (HMT) and Edge-aware Local Transformer (ELT), which is supplemented with adaptive contextual refinement (ACR), a two-stage attention network (TAN), and a meta-ensemble classifier for decision stability. The model demonstrated commendable performance, achieving over 99% in accuracy, precision, recall, and F1-score across four benchmark mammography datasets: CBIS-DDSM, DDSM, INBreast, and MIAS. The integration of explainable artificial intelligence techniques such as Grad-CAM, SHAP, and OSA provides effective visual elucidations for forecasts that closely align with preliminary interpretability assessment.

The endeavor is hindered by two primary obstacles. The model was tested exclusively on public datasets; its performance in real clinical variability, influenced by various imaging hardware and demographic populations, remains unevaluated. Secondly, the model does not provide a quantitative comparison with expert-annotated lesion maps to thoroughly assess the saliency outputs despite the inclusion of visual explanations. Future studies will concentrate on various perspectives. This involves the integration of multi-view mammography (CC and MLO) fusion to enhance diagnostic performance and external validation using multi-institutional clinical datasets to assess generalization across many clinical scenarios. Efforts will concentrate on developing radiologist-in-the-loop frameworks that integrate human expertise with artificial intelligence-driven decision support to enhance the safety and reliability of diagnostic systems. Additionally, quantitative metrics will be established to assess explanation quality and optimize model architecture for deployment on edge devices. Although the proposed TIxAI framework provides a preliminary quantitative assessment of explanation consistency, future studies incorporating expert radiologist annotations and clinically validated lesion masks are required to establish stronger clinical interpretability and trustworthiness.

## Figures and Tables

**Table 1:** Feature transformation pipeline of the proposed STARE-Net framework.

Stage	Operation	Output Representation
Input	Mammogram Image	H × W × 1
Preprocessing	Normalization + Gaussian Filtering	H × W × 1
Backbone	MobileNetV2 Feature Extraction	C × H/16 × W/16
HMT	Multi-Scale Global Attention	C × H/4 × W/4
ELT	Edge-Aware Local Attention	C × H/2 × W/2
ACR	Contextual Feature Refinement	C × H × W
GAP	Global Average Pooling	C
MLP	Feature Projection Layer	d
MEC	WSVM + KNN Classification	Class Probabilities

**Table 2:** Dataset label harmonization and class distribution summary.

Dataset	Original Labels	Normal	Benign	Malignant	Removed Images
CBIS-DDSM	Benign, Malignant	–	891	637	Duplicate/Corrupted Only
DDSM	Normal, Benign, Malignant	695	780	1135	Duplicate/Corrupted Only
INBreast	Normal, Benign, Malignant	107	107	196	Duplicate/Corrupted Only
MIAS	Normal, Abnormal	208	63	51	Duplicate/Corrupted Only

**Table 3:** Performance and parameter efficiency comparison of different deep learning architectures across each dataset.

Dataset	Model	Accuracy (%)	Precision (%)	Recall (%)	F1-Score (%)	Kappa	Params (M)
CBIS-DDSM	EfficientNetB4	82.1	81.5	81.8	81.6	0.65	19.0
	InceptionV2	78.4	77.8	78.0	77.9	0.60	8.8
	ViT (Base/16)	88.5	88.0	88.2	88.1	0.80	86.6
	Swin-T	92.3	91.8	92.0	91.9	0.88	28.3
	STARE-Net	99.2	98.7	98.9	99.1	0.97	11.3
DDSM	EfficientNetB4	80.5	80.0	80.2	80.1	0.63	19.0
	InceptionV2	75.3	74.8	75.0	74.9	0.58	8.8
	ViT (Base/16)	87.0	86.5	86.7	86.6	0.78	86.6
	Swin-T	91.5	91.0	91.2	91.1	0.86	28.3
	STARE-Net	99.3	98.9	99.0	99.2	0.98	11.3
INBreast	EfficientNetB4	79.2	78.7	78.9	78.8	0.60	19.0
	InceptionV2	74.1	73.5	73.8	73.6	0.55	8.8
	ViT (Base/16)	86.7	86.2	86.4	86.3	0.75	86.6
	Swin-T	90.8	90.3	90.5	90.4	0.82	28.3
	STARE-Net	99.4	99.1	99.2	99.3	0.99	11.3
MIAS	EfficientNetB4	84.0	83.5	83.7	83.6	0.70	19.0
	InceptionV2	72.4	72.0	72.2	72.1	0.52	8.8
	ViT (Base/16)	85.9	85.4	85.6	85.5	0.74	86.6
	Swin-T	89.6	89.1	89.3	89.2	0.80	28.3
	STARE-Net	99.1	98.5	98.7	99.0	0.97	11.3

**Table 4:** Performance of models on different datasets with and without preprocessing.

Dataset	Preprocessing	Accuracy (%)	Precision (%)	Recall (%)	F1-Score (%)	Kappa
CBIS-DDSM	Yes	99.2	98.7	98.9	99.1	0.97
	No	97.5	96.8	96.9	97.2	0.94
DDSM	Yes	99.3	98.9	99.0	99.2	0.98
	No	97.8	97.1	97.2	97.5	0.95
INBreast	Yes	99.4	99.1	99.2	99.3	0.99
	No	98.1	97.3	97.5	97.7	0.96
MIAS	Yes	99.1	98.5	98.7	99.0	0.97
	No	96.9	96.2	96.3	96.5	0.93

**Table 5:** Validation performance of backbone architectures across each dataset.

Dataset	Metric	ResNet50	DenseNet121	InceptionV3	EfficientNet-B0	(Proposed)
CBIS-DDSM	Accuracy (%)	98.7	98.9	98.5	99.0	99.2
	F1 Score (%)	98.5	98.7	98.2	98.8	99.1
	Kappa	0.96	0.97	0.95	0.97	0.97
DDSM	Accuracy (%)	98.8	99.0	98.6	99.1	99.3
	F1 Score (%)	98.6	98.8	98.3	98.9	99.2
	Kappa	0.97	0.97	0.96	0.98	0.98
INBreast	Accuracy (%)	98.9	99.1	98.8	99.2	99.4
	F1 Score (%)	98.7	99.0	98.6	99.1	99.3
	Kappa	0.97	0.98	0.97	0.98	0.99
MIAS	Accuracy (%)	97.6	98.0	97.4	98.5	99.1
	F1 Score (%)	97.2	97.6	97.0	98.1	99.0
	Kappa	0.94	0.95	0.94	0.96	0.97

**Table 6:** Performance of ensemble techniques on different datasets.

Dataset	Metric	Bagging	Boosting	Stacking	WMV (Proposed)
CBIS-DDSM	Accuracy (%)	98.9	99.1	99.3	99.2
	F1 Score (%)	98.7	98.9	99.2	99.1
	Kappa	0.96	0.97	0.98	0.97
DDSM	Accuracy (%)	99.0	99.2	99.4	99.3
	F1 Score (%)	98.8	99.0	99.2	99.2
	Kappa	0.97	0.98	0.99	0.98
INBreast	Accuracy (%)	99.2	99.4	99.5	99.4
	F1 Score (%)	99.0	99.2	99.3	99.3
	Kappa	0.98	0.99	0.99	0.99
MIAS	Accuracy (%)	98.0	98.3	98.7	99.1
	F1 Score (%)	97.8	98.1	98.4	99.0
	Kappa	0.95	0.96	0.97	0.97

**Table 7:** Cross-dataset performance evaluation for ensemble techniques.

Training Dataset	Testing Dataset	Accuracy (%)	F1 Score (%)	Precision (%)	Recall (%)	Kappa
CBIS-DDSM	CBIS-DDSM	99.2	99.1	99.3	99.1	0.97
	DDSM	98.8	98.6	98.7	98.5	0.96
	INBreast	99.0	98.9	99.1	98.7	0.97
	MIAS	98.6	98.4	98.5	98.3	0.95
DDSM	CBIS-DDSM	98.7	98.5	98.8	98.6	0.95
	DDSM	99.3	99.2	99.3	99.0	0.98
	INBreast	99.1	99.0	99.2	99.0	0.97
	MIAS	98.5	98.3	98.4	98.1	0.94
INBreast	CBIS-DDSM	99.3	99.2	99.4	99.1	0.98
	DDSM	99.0	98.9	99.1	98.9	0.96
	INBreast	99.5	99.4	99.6	99.4	0.99
	MIAS	99.0	98.8	99.1	98.9	0.97
MIAS	CBIS-DDSM	98.9	98.7	98.9	98.7	0.96
	DDSM	98.7	98.5	98.7	98.6	0.95
	INBreast	99.1	99.0	99.2	99.0	0.97
	MIAS	99.1	99.0	99.2	99.0	0.97

**Table 8:** Statistical robustness analysis of the proposed STARE-Net framework across repeated runs.

Dataset	Accuracy (%)	Std. Dev	95% CI
CBIS-DDSM	99.21 ± 0.18	0.18	[98.95, 99.47]
DDSM	99.08 ± 0.22	0.22	[98.76, 99.40]
INBreast	99.34 ± 0.16	0.16	[99.11, 99.57]
MIAS	99.12 ± 0.21	0.21	[98.82, 99.42]

**Table 9:** McNemar statistical significance analysis against baseline models.

Baseline Model	Dataset	*p*-Value
Swin-T	CBIS-DDSM	0.013
ViT	CBIS-DDSM	0.009
EfficientNetB4	DDSM	0.017
InceptionV3	INBreast	0.011

**Table 10:** Effect of each module on classification performance.

Dataset	Metric	Backbone	+HMT	+ELT	+TAN	+TAN+ACR	+TAN+ACR+MEC
CBIS-DDSM	Accuracy (%)	74.5	89.0	91.2	96.1	99.1	99.2
	F1 Score (%)	74.0	89.3	91.5	96.3	99.0	99.1
	Kappa	0.74	0.89	0.91	0.96	0.96	0.97
DDSM	Accuracy (%)	78.0	89.7	91.1	96.5	99.2	99.3
	F1 Score (%)	77.5	89.9	91.3	96.7	99.1	99.2
	Kappa	0.78	0.89	0.91	0.97	0.97	0.98
INBreast	Accuracy (%)	72.0	88.5	90.7	95.2	99.3	99.4
	F1 Score (%)	71.8	88.7	90.9	95.4	99.2	99.3
	Kappa	0.72	0.88	0.90	0.95	0.98	0.99
MIAS	Accuracy (%)	79.5	89.2	91.6	95.7	99.0	99.1
	F1 Score (%)	79.0	89.4	91.8	95.9	98.9	99.0
	Kappa	0.79	0.89	0.92	0.96	0.96	0.97

**Table 11:** Performance comparison of the proposed STARE-Net method with state-of-the-art models on multiple datasets.

Reference	Dataset	Accuracy (%)	Precision (%)	Recall (%)	F1-Score (%)
Hybrid DL [22]	CBIS-DDSM	96.0	95.0	95.0	–
Multi-patch DCAE + VGG19 [24]	CBIS-DDSM	98.3	97.9	94.8	–
STARE-Net (Proposed)	CBIS-DDSM	99.2	98.7	98.9	99.1
Stacking-Enhanced Bagging ensemble CNN [21]	DDSM	98.8	–	94.8	94.1
Customized AlexNet and SVM [43]	DDSM	99.16	–	97.13	–
STARE-Net (Proposed)	DDSM	99.3	98.9	99.0	99.2
Ensemble of VGG16, DenseNet121, InceptionV3 [18]	INBreast	90.1	–	88.3	89.1
BreastNet-SVM pipeline [19]	INBreast	99.16	–	97.13	–
STARE-Net (Proposed)	INBreast	99.4	99.1	99.2	99.3
Multi-patch DCAE + VGG19 [24]	MIAS	98.95	97.99	97.20	–
TF-based DL model [29]	MIAS	96.55	–	–	–
STARE-Net (Proposed)	MIAS	99.1	99.5	98.7	99.0

## Data Availability

The datasets utilized in this paper are publicly available at Digital Database for Screening Mammography (DDSM) [39], Curated Breast Imaging Subset (CBIS-DDSM) [40], INBreast [41], and Mammographic Image Analysis Society (MIAS) [42]. The implementation of this paper is available at https://github.com/Armughan-Ali/STARE-NET (accessed on 27 May 2026).
